# Bronchial Atresia Associated With a Giant Subpleural Bulla: A Case Report

**DOI:** 10.7759/cureus.117874

**Published:** 2026-10-13

**Authors:** Carolina P Figueira, Helena Fragoeiro, Maria Cunha, Joana Lobato, Bruno F Couto, Mariana P Afonso

**Affiliations:** 1 General and Family Medicine, Câmara de Lobos Health Center, Serviço de Saúde da Região Autónoma da Madeira, Entidade Pública Empresarial da Região Autónoma da Madeira (SESARAM, EPERAM), Câmara de Lobos, PRT; 2 General and Family Medicine, Ribeira Brava Health Center, Serviço de Saúde da Região Autónoma da Madeira, Entidade Pública Empresarial da Região Autónoma da Madeira (SESARAM, EPERAM), Ribeira Brava, PRT; 3 General and Family Medicine, Arco da Calheta Health Center, Serviço de Saúde da Região Autónoma da Madeira, Entidade Pública Empresarial da Região Autónoma da Madeira (SESARAM, EPERAM), Calheta, PRT; 4 General and Family Medicine, Estreito de Câmara de Lobos Health Center, Serviço de Saúde da Região Autónoma da Madeira, Entidade Pública Empresarial da Região Autónoma da Madeira (SESARAM, EPERAM), Câmara de Lobos, PRT

**Keywords:** bronchial atresia, bullous disease, giant pulmonary bulla, iatrogenic aortic injury, video-assisted thoracoscopic surgery

## Abstract

Bronchial atresia is a rare congenital malformation characterized by focal interruption of a lobar, segmental, or subsegmental bronchus, with distal mucus impaction and hyperinflation of the affected lung. It is usually asymptomatic and detected incidentally. We report the case of a 25-year-old man with no relevant medical history who presented to primary care with a three-day history of productive cough, rhinorrhea, nasal congestion, and myalgia. Physical examination revealed hyperresonance to percussion and markedly diminished breath sounds throughout the left hemithorax. Chest radiography showed marked left-sided hyperlucency with contralateral mediastinal shift. Chest computed tomography demonstrated a focal abnormality of the left upper lobe bronchus with distal mucus-filled bronchial dilatation consistent with a bronchocele, together with a giant subpleural bulla measuring approximately 25 cm along its longest axis and occupying about half of the left hemithorax. After multidisciplinary assessment, elective video-assisted thoracoscopic resection of the bulla was performed. The procedure was complicated by an iatrogenic laceration of the descending aorta during division of the inferior pulmonary ligament, causing major arterial hemorrhage and requiring emergent conversion to thoracotomy and direct aortic repair. The postoperative course was complicated by left pleural empyema, which was successfully treated with antibiotics and drainage. At three months, the patient reported only mild exertional dyspnea, with no recurrent respiratory infection or pneumothorax and satisfactory radiographic re-expansion of the remaining left lung. This case highlights an uncommon association between bronchial atresia and extensive bullous disease, the value of careful assessment of disproportionate physical findings, and the importance of individualized surgical planning when marked intrathoracic anatomical distortion is present.

## Introduction

Bronchial atresia is a rare congenital anomaly of the tracheobronchial tree, characterized by focal interruption of a bronchus, with a male predominance (male-to-female ratio of 2:1) and an estimated prevalence of 1.2 cases per 100,000 males [1]. The distal airway remains patent and fills with secretions, producing a bronchocele or mucocele, while collateral ventilation through adjacent airways leads to air trapping and hyperinflation of the distal lung parenchyma, which, in rare cases, may progress to emphysema and bulla formation [2-5]. Most patients (approximately two-thirds) are asymptomatic, and the diagnosis is usually incidental. When present, symptoms include recurrent respiratory infections, chronic cough, fever, respiratory distress, and spontaneous pneumothorax [1].

Computed tomography (CT) is central to diagnosis. The characteristic radiologic pattern combines a mucus-filled bronchial structure with hyperinflation and reduced vascularity of the adjacent lung, and the left upper lobe is a commonly involved site [2,6].

We describe a young adult with radiologic findings consistent with bronchial atresia associated with a giant subpleural bulla occupying approximately half of the left hemithorax. The patient underwent elective thoracoscopic resection, which was complicated by an iatrogenic descending aortic injury requiring emergent thoracotomy.

## Case presentation

A 25-year-old man who worked as a mechanic and had no relevant personal or family medical history, no known drug allergies, and no reported substance use presented to primary care with a three-day history of productive cough, anterior rhinorrhea, nasal congestion, and myalgia. He showed no signs of respiratory distress and was hemodynamically stable. Pulmonary examination was notable for hyperresonance to percussion and markedly diminished breath sounds throughout the left hemithorax.

Because the physical findings were disproportionate to the mild acute respiratory symptoms, he was referred to the emergency department for further investigation. Chest radiography demonstrated marked hyperlucency of the left hemithorax with contralateral mediastinal shift (Figure 1). Chest CT subsequently showed a focal abnormality of the left upper lobe bronchus suggestive of bronchial atresia, associated with peripheral bronchiectatic changes, some of which were mucus-filled and formed a bronchocele. A giant subpleural bulla was present in the left upper lobe, measuring approximately 25 cm in longitudinal diameter, compressing the ipsilateral lower lobe and displacing the mediastinum to the right (Figure 2). Alpha-1 antitrypsin testing showed no deficiency.

**Figure 1:**
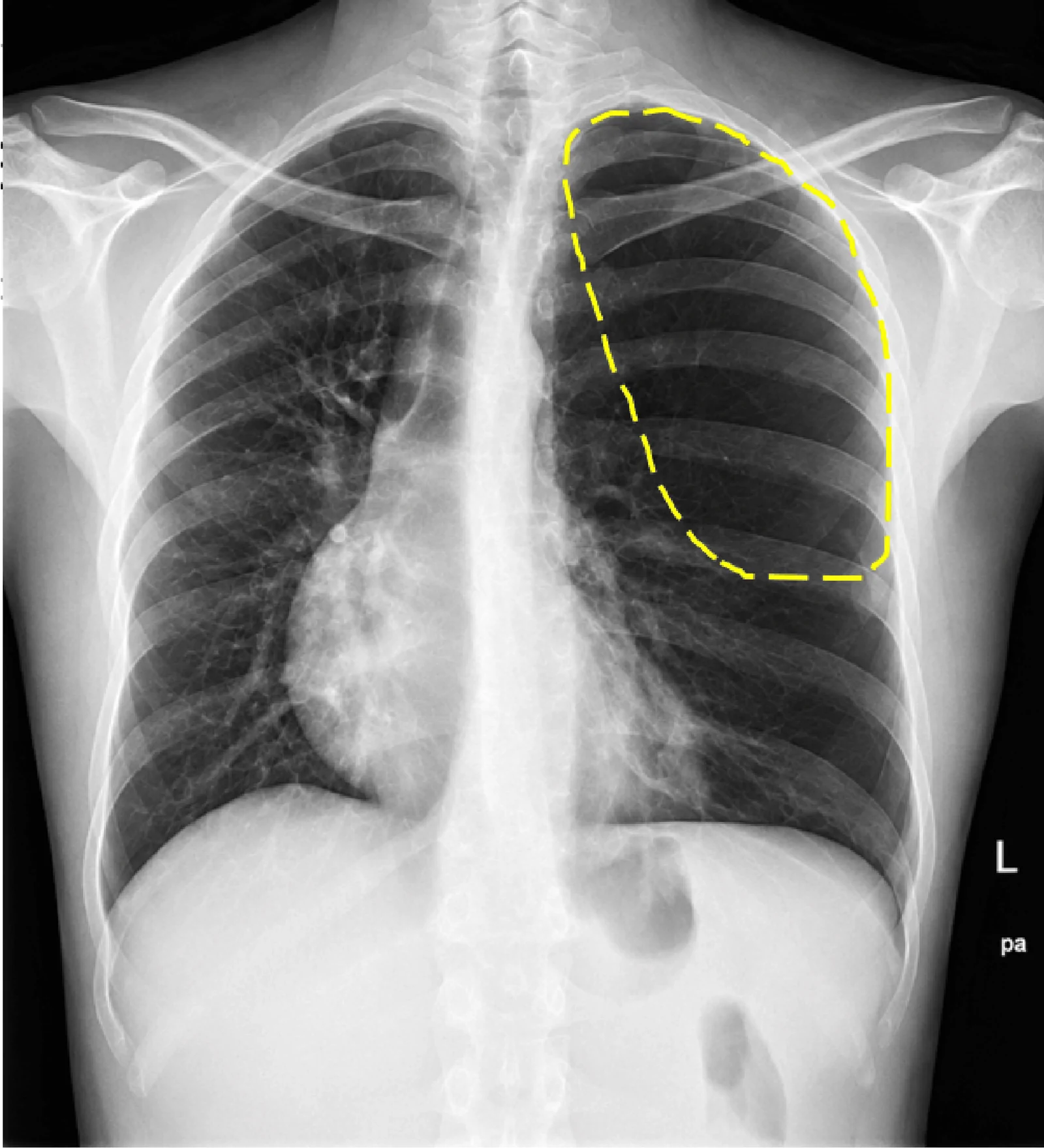
Posteroanterior initial chest radiograph Posteroanterior chest radiograph obtained during the initial evaluation, showing marked hyperlucency of the left hemithorax (dashed yellow outline) with contralateral mediastinal shift.

**Figure 2:**
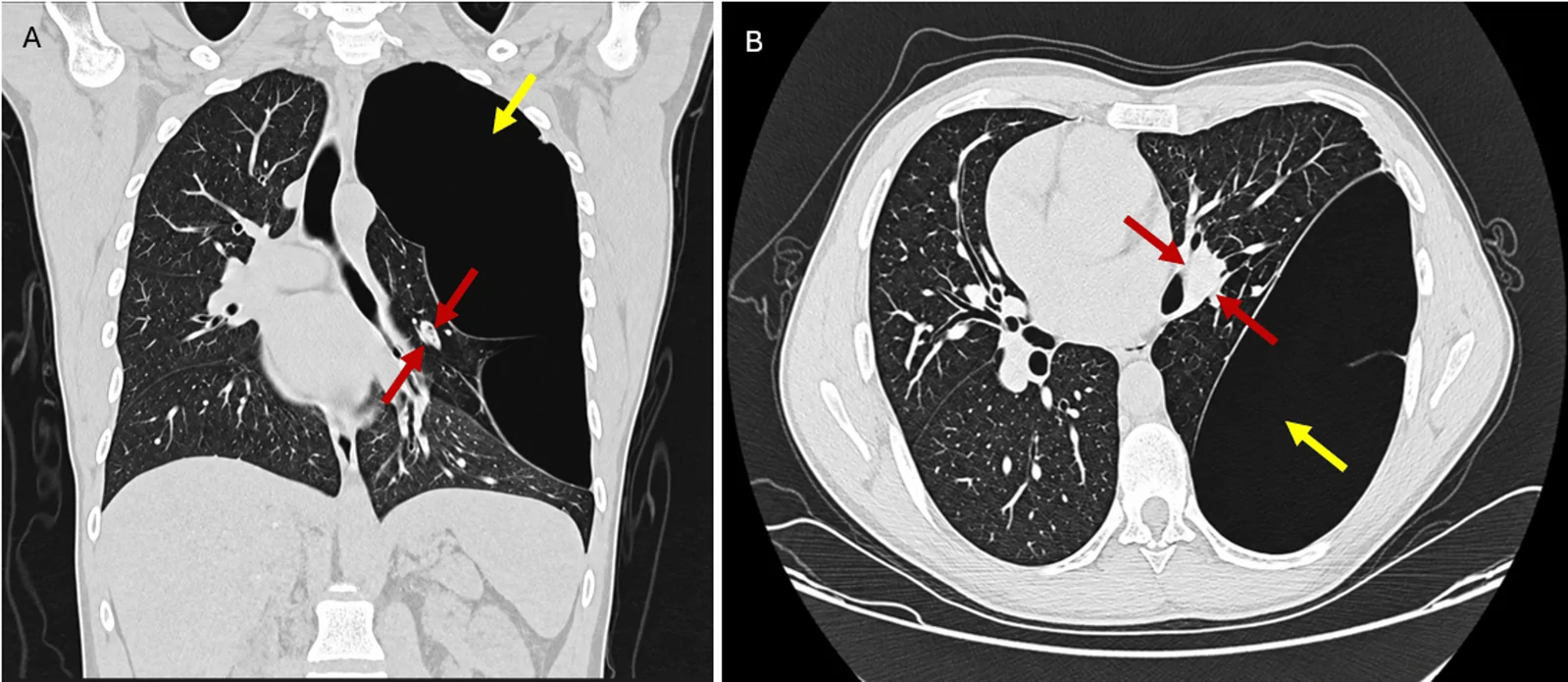
Giant subpleural bulla and bronchocele on chest CT Coronal (A) and axial (B) chest CT images showing a giant subpleural bulla in the left upper lobe (yellow arrow) and a mucus-filled blind-ending bronchus/bronchocele (red arrows).

The case was discussed by Pulmonology and Cardiothoracic Surgery. Given the patient's clinical stability and the absence of symptoms clearly attributable to the structural lesion, he was initially discharged with warning signs and scheduled for multidisciplinary outpatient follow-up. Because of the size of the bulla, its substantial compressive effect on the remaining lung, and the associated mediastinal displacement, elective surgical treatment was subsequently proposed.

Video-assisted thoracoscopic surgery (VATS) was performed two months after diagnosis, with wedge resection of the left upper lobe at the base of the bulla; the resection was confined to the bulla, and no anatomical segmentectomy or lobectomy directed at the atretic bronchial segment was undertaken. During division of the inferior pulmonary ligament to facilitate mobilization and re-expansion of the remaining lung, an iatrogenic laceration of the descending aorta occurred, resulting in major arterial hemorrhage. The procedure was immediately converted to an open thoracotomy. Volume resuscitation with intravenous fluids and blood products was instituted, and the aortic laceration was repaired by direct suture, with a maximum total aortic clamping time of 12 minutes. The patient was admitted to the intensive care unit for two days after surgery, requiring vasopressor support.

During the hospital stay, the patient developed bilateral pneumonia and left pleural empyema (Figures 3, 4), managed with pleural drainage and antibiotic therapy. Empirical meropenem was administered for three days. Pleural fluid culture yielded Streptococcus mitis/oralis, and therapy was de-escalated according to the susceptibility profile of the isolate to intravenous clindamycin plus gentamicin, completed over nine days, followed by oral clindamycin for a further two weeks after discharge. Histopathologic examination of the surgical specimen confirmed a subpleural emphysematous bulla, together with numerous bronchiectatic and bronchiolectatic changes containing abundant mucus. Clinical evolution was subsequently favorable, the chest drain was removed 17 days after surgery, and the patient was discharged after 22 days of hospitalization.

**Figure 3:**
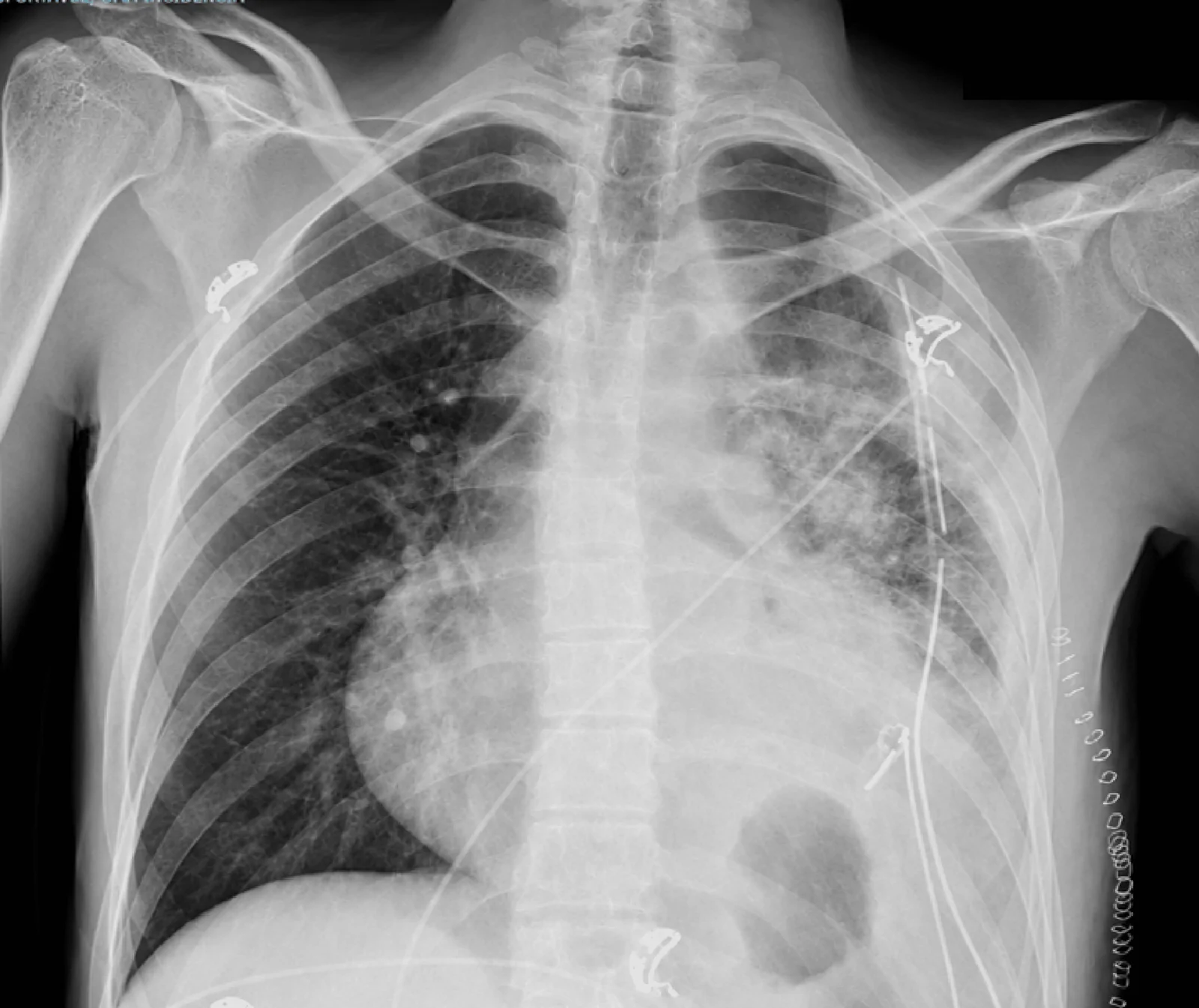
Postoperative pneumonia and pleural empyema Postoperative chest radiograph demonstrating left lower lobe pneumonia and pleural empyema.

**Figure 4:**
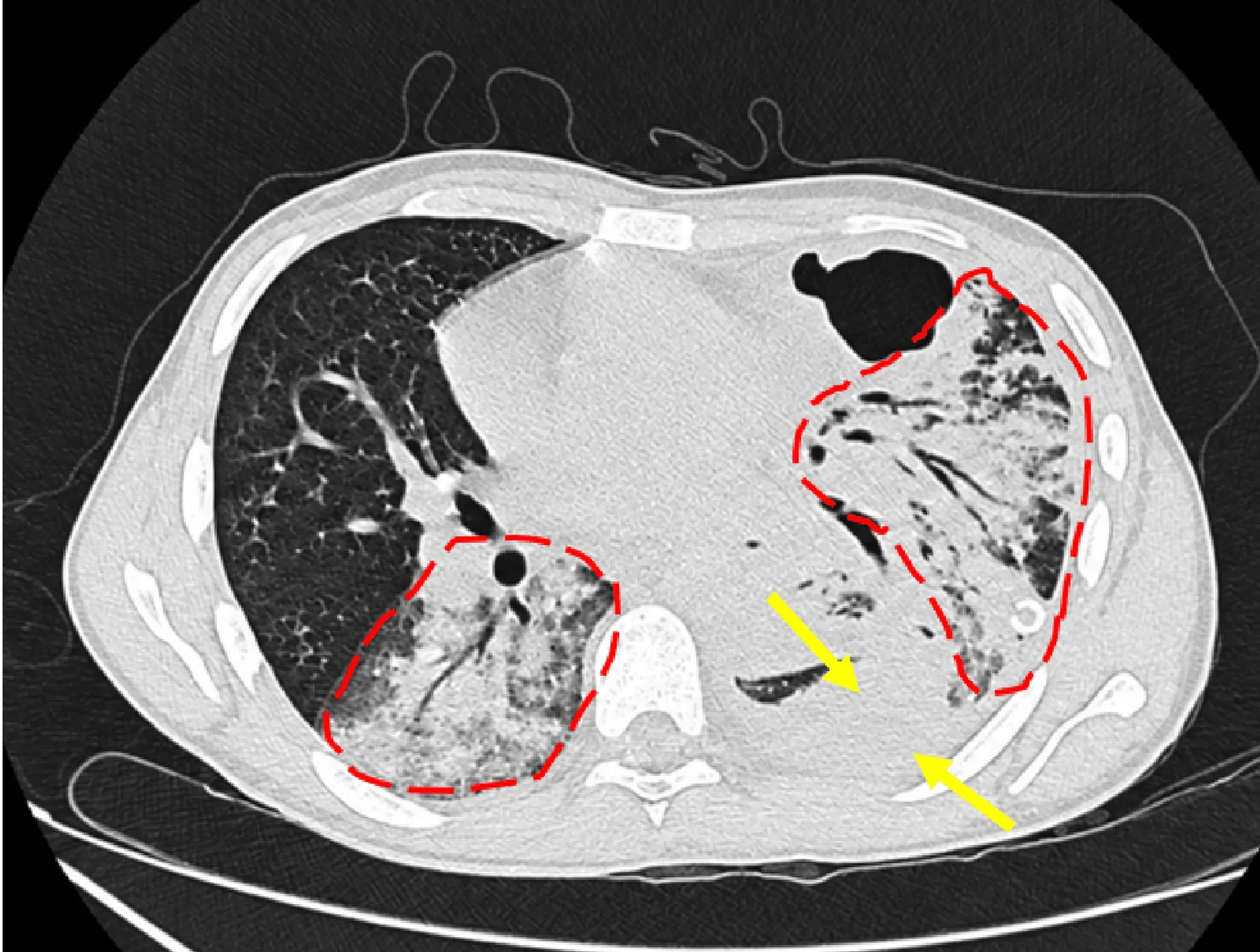
Postoperative pleural empyema and bilateral lower lobe pneumonia Axial postoperative chest CT image demonstrating left pleural empyema (yellow arrows) and bilateral lower lobe pneumonia (red dashed line).

At the three-month follow-up, the patient reported mild dyspnea on exertion, which had persisted since discharge, with no recurrent respiratory infections, no pneumothorax, and no further surgery-related complications. He was awaiting the start of a pulmonary rehabilitation program. Follow-up chest X-ray demonstrated satisfactory re-expansion of the remaining left lung (Figure 5).

**Figure 5:**
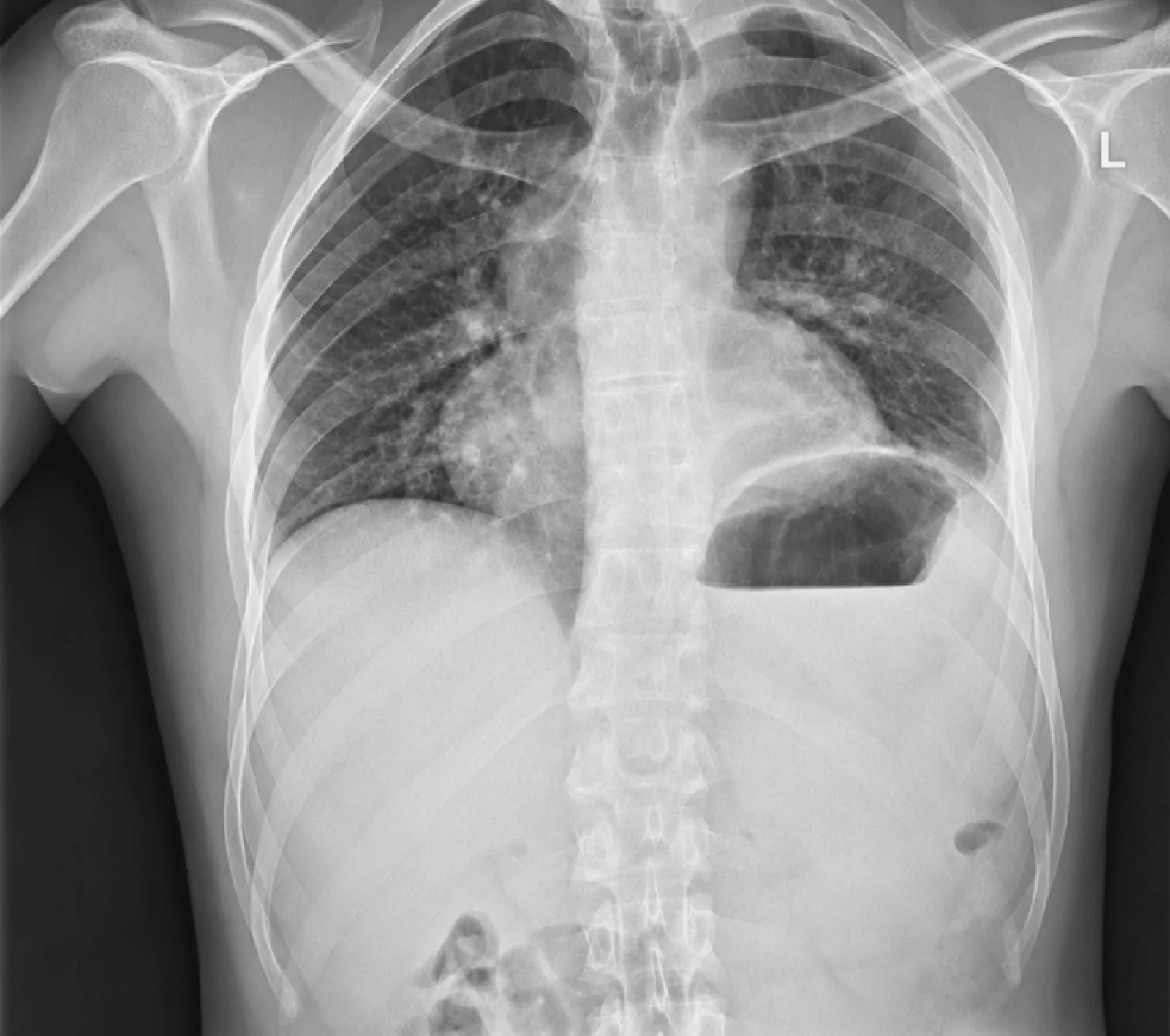
Three-month follow-up lung re-expansion Three-month follow-up posteroanterior chest radiograph showing satisfactory re-expansion of the remaining left lung.

## Discussion

Bronchial atresia is typically a benign congenital airway anomaly and is often discovered incidentally in otherwise healthy young adults. The interruption of the affected bronchus leads to distal mucus retention, while collateral ventilation maintains aeration of the obstructed segment and produces localized air trapping and hyperinflation [2,3,6]. In the present case, CT demonstrated a focal left upper lobe bronchial abnormality with mucus-filled peripheral bronchial dilatation and marked ipsilateral hyperinflation, a radiologic constellation considered characteristic of bronchial atresia.

A pulmonary bulla is commonly considered giant when it occupies at least one-third of a hemithorax [7]. The lesion in this patient measured approximately 25 cm along its longest axis and occupied about half of the left hemithorax, with compression of the remaining ipsilateral lung and contralateral mediastinal shift. Previous reports have described subpleural bullae and spontaneous pneumothorax in lung segments distal to an atretic bronchus [4,5]. Chronic overinflation and mechanical stress on distal alveolar structures provide a plausible explanation for such bullous changes, although a direct causal relationship cannot be established from a single case. Bullae of the magnitude observed here in association with bronchial atresia appear to be exceptionally uncommon.

Management of bronchial atresia is not standardized and should be individualized according to symptoms, recurrent infection, associated complications, and the degree of parenchymal or anatomical compromise. For giant pulmonary bullae, VATS can achieve effective resection and functional improvement, although postoperative complications, particularly prolonged air leak, are well recognized [8]. Thoracoscopic approaches are also increasingly used in selected adults with bronchial atresia, including sublobar resections and lobectomy, and recent reports emphasize the value of careful preoperative anatomical assessment [9-11]. In this patient, despite the absence of chronic symptoms attributable to the lesion, the extreme size of the bulla, compression of the remaining left lung, and mediastinal displacement supported elective surgical treatment.

The most serious event in this case was iatrogenic injury to the descending aorta during thoracoscopic dissection. Major vascular injury during VATS is uncommon but potentially life-threatening and may require immediate conversion to thoracotomy for definitive control [11]. The severe anatomical distortion caused by the giant bulla may have increased the technical complexity of the procedure; however, this remains a plausible contributing factor rather than a demonstrated cause of the aortic injury. The event underscores the importance of detailed preoperative imaging, anticipation of altered anatomy, and a low threshold for conversion when major bleeding occurs.

The postoperative empyema represented a second major complication but resolved with antibiotic therapy and drainage. From a diagnostic perspective, the case also illustrates an important primary care principle: mild or nonspecific presenting symptoms should not override markedly abnormal physical findings. The pronounced unilateral hyperresonance and reduction in breath sounds prompted imaging and led to the recognition of a clinically silent but anatomically significant thoracic abnormality.

This report has some limitations. Bronchoscopy, pulmonary function tests, and previous thoracic imaging were not available, which limited direct characterization of the atretic bronchus, objective assessment of the functional impact of the bulla and its resection, and determination of the time course of bulla development.

## Conclusions

Bronchial atresia is usually an incidental and clinically benign congenital anomaly, but chronic distal hyperinflation may be associated with substantial structural lung changes. In this young patient, radiologic findings consistent with bronchial atresia coexisted with a giant subpleural bulla occupying approximately half of the left hemithorax, compressing the remaining lung and producing mediastinal displacement. The case demonstrates the value of CT for simultaneous characterization of the airway abnormality, bullous disease, and their anatomical consequences, as well as the importance of acting on physical examination findings that are disproportionate to the presenting symptoms.

Therapeutic decisions in uncommon and anatomically complex presentations should be individualized and multidisciplinary. Although a minimally invasive approach may be appropriate for giant pulmonary bullae, severe distortion of intrathoracic anatomy can increase operative complexity, and major vascular injury remains a potentially catastrophic complication requiring prompt recognition and conversion to open surgery when necessary. Careful preoperative planning, appropriate surgical preparedness, and coordinated postoperative follow-up are therefore essential in selected patients with bronchial atresia complicated by extensive bullous disease.
